# Pathophysiology of Clopidogrel in Ischemic Stroke, Role of Platelet microRNAs

**DOI:** 10.33549/physiolres.935617

**Published:** 2025-08-01

**Authors:** Tomáš MIČANÍK, Ondřej SLABÝ, Tomáš KVASNIČKA, Petra BOBČÍKOVÁ, Jana VAVROŠOVÁ, Eva AUGSTE, Daniel VÁCLAVÍK

**Affiliations:** 1 Department of Physiology and Pathophysiology, Faculty of Medicine, University of Ostrava, Ostrava, Czech Republic; 2 Department of Physiology and Pathophysiology, First Faculty of Medicine, Charles University, Prague, Czech Republic; 3 Central European Institute of Technology (CEITEC), Masaryk University, Brno, Czech Republic; 4 Centre for Thrombosis, General University Hospital in Prague, Prague, Czech Republic; 5 Department of Anaesthesiology, Resuscitation and Intensive Care, Faculty of Medicine, University of Ostrava, Ostrava, Czech Republic; 6 Department of Neurology, AGEL Hospital Ostrava-Vítkovice, Ostrava, Czech Republic

**Keywords:** CYP2C19, microRNA, Clopidogrel, Ischemic stroke, Antiplatelet therapy

## Abstract

Variation in response to clopidogrel represents a significant clinical challenge in patients with ischemic stroke. Genetic polymorphisms cytochrome P450 2C19 (CYP2C19) are a known cause of resistance to clopidogrel. Platelet microRNAs (miRNAs) can modulate the efficacy of antiplatelet therapy. This study focuses solely on clopidogrel because it is the most widely used alternative to aspirin in patients with aspirin intolerance or contraindications. Our aim was to investigate its pharmacogenomic and epigenetic modulation in a targeted and homogeneous cohort. CYP2C19 genotypes are commonly reported as *1/*1 (wild type), *1/*2 (intermediate metabolizer), *2/*2 (poor metabolizer) and *2/*3 (poor metabolizer). These denote the number and type of loss-of-function alleles that affect clopidogrel metabolism. Clopidogrel treatment is typically a component of broader secondary prevention strategies, including lifestyle modifications, statins, and control of blood pressure. Relevant bibliographic references have been added to support the background statements provided in the introduction and methodology. To evaluate the expression of selected platelet miRNAs (miR-126-3p, miR-19a-3p, miR-19b-3p, miR-22-3p, miR-185-5p) in patients with ischemic stroke in relation to the CYP2C19 genotype (*1/*1,*1/*2, *2/*2) during clopidogrel treatment. Seventy patients treated with clopidogrel (75 mg daily) were enrolled. Patients were genotyped for the CYP2C19 *2 and *3 alleles by real-time polymerase chain reaction (polymerase chain reaction (PCR)) and miRNA expression was measured in plasma. All abbreviations used throughout the manuscript have been defined at their first appearance for the sake of clarity. No significant differences in miRNA expression were found between the genotypic groups (p > 0.05). Patients with genotype *2/*2 (poor metabolizer) showed a trend towards higher levels of miR-126-3p and miR-185-5p (approximately 1.5 to 1.7 times) compared to *1/*1 (wild type). The clinical parameters did not differ significantly between the groups. Poor clopidogrel metabolizers can exhibit upregulation of some platelet miRNAs as a potential compensatory mechanism. This pilot study suggests a possible epigenetic modulation of the response to antiplatelet therapy through platelet miRNAs.

## Introduction

Ischemic Clopidogrel is a prodrug activated by liver enzymes of cytochrome P450, predominantly the CYP2C19 isoenzyme. Polymorphisms in the CYP2C19 gene, such as allele variants *2 and *3, which lead to loss of function, and *17, which confers increased enzymatic activity, result in variable drug metabolism. Loss of function variants causes reduced or absent enzyme activity and can lead to resistance to clopidogrel. On the contrary, gain-of-function variants such as *17 may increase the risk of bleeding. Patients with a poor metabolizer genotype (*2/*2) may be at increased risk for recurrent ischemic events due to insufficient platelet inhibition.

Stroke is one of the leading causes of morbidity and mortality worldwide. In patients after ischemic stroke, it is standard practice to initiate secondary prophylaxis with antiplatelet therapy. In case of aspirin intolerance, an alternative P2Y_12_ receptor inhibitor, clopidogrel, is most often used. In recent years, attention has focused on the role of microRNAs (miRNAs), short noncoding RNA molecules, in the regulation of platelet functions.

Several miRNAs (for example, miR-126-3p, miR-19a-3p, miR-19b-3p, miR-22-3p, miR185-5p) have been reported to be regulators of platelet receptor expression, signaling pathways, and platelet aggregation [1–3]. Their expression can be influenced by genetic factors and may represent an additional layer of regulation in response to antithrombotic therapy. However, the association between CYP2C19 polymorphisms and platelet miRNA expression has not been adequately explored in the context of ischemic stroke. In this study, our objective was to evaluate the expression of selected platelet miRNAs (miR-126-3p, miR-19a-3p, miR-19b-3p, miR-22-3p, miR-185-5p) in patients with ischemic stroke who receive clopidogrel according to their genotypic status of CYP2C19 (*1/*1, *1/*2, *2/*2).

## Methods

### Study population

A total of 70 patients with ischemic stroke were included in the study. All patients were treated with 75 mg of clopidogrel daily as part of secondary prevention. In each patient, the CYP2C19 poly-morphisms were genotyped and the expression of selected platelet miRNAs was analyzed. Only blood samples without evidence of hemolysis (defined as ΔCq (miR-23a – miR-451a) > 7) were included in the analysis to ensure sample quality. Although the study initially included 70 patients, genotype-based analysis of miRNA expression was conducted in a subset of 59 individuals, based on the presence of the most frequent genotypes (*1/*1, *1/*2, *2/*2). Patients with other rare genotypes or incomplete data were excluded from this part of the analysis.

Among the 70 patients, there were 6 poor metabolizers with genotype *2/*2 (8.6 %), 9 intermediate metabolizers with genotype *1/*2 (12.9 %), and the rest had wild-type genotype *1/*1 or other genotypic variants.

### Genotyping of CYP2C19

Alleles CYP2C192 (rs4244285) and CYP2C193 (rs4986893) were determined by realtime PCR with melting curve analysis, using fluorescent hybridization probes (HybProbe and SimpleProbe probes, TIB MOLBIOL, Roche). Genomic deoxyribonucleic acid (DNA) was isolated from EDTA-anticoagulated blood using a magnetic bead method

(MagCore® HF16, RBC Bioscience). PCR amplification and melting curve analysis were performed on a LightCycler® Cobas z480 according to the manufacturer’s protocols (LightMix® kit CYP2C19*2, *3).

### Analysis of miRNA expression

The expression of selected platelet miRNAs (hsa-miR-19a-3p, hsa-miR-19b-3p, hsa-miR-22-3p, hsa-miR-126-3p, hsa-miR-185-5p) was quantified using a bilaterally primed reverse transcription quantitative polymerase chain reaction (RT-qPCR) method (BioVendor). Total RNA was isolated from platelet-poor plasma using the miRCURY RNA isolation kit (Qiagen). The efficiency of RNA isolation was monitored using synthetic RNA spike-in controls and the quality of RNA was verified on the Fragment Analyzer (Agilent) system. Reverse transcription and quantitative PCR were performed on a CFX384 real-time PCR system (Bio-Rad).

### Quality control of RNA isolation

To ensure reliable results, additional quality control measures were implemented during RNA isolation. Synthetic spike-in controls (eg. cel-miR-39-3p) were added to each sample prior to isolation to monitor the efficiency and consistency of the process. Furthermore, the integrity and purity of isolated RNA was assessed using the Fragment Analyzer system. Samples exhibiting signs of degradation or contamination were excluded from further analysis. Samples showing hemolysis were excluded from further analysis as described above.

### Statistical analysis

Statistical analysis was performed in R (version 4.3.3; R Core Team, 2024). Relative levels of miRNA expression were calculated using the 2^^−ΔΔCq^ comparative method (using the genotype group *1/*1 as a reference). For group comparisons, the Kruskal-Wallis test was used for nonparametric data. Furthermore, univariate linear regression (model: miRNA ~ genotype) was used to assess the relationship between genotype (normally considered alleles 0, 1 or 2 *2) and miRNA expression. Before applying these tests, basic assumptions (normality of distribution and homogeneity of variances) were verified. A p-value < 0.05 was considered statistically significant.

The study was approved by the Ethics Committee of Vitkovice Hospital (Ostrava, CZ) under reference number EK35/2019 and was carried out in accordance with the principles of the Declaration of Helsinki. All participants gave their informed consent in writing before enrolling in the study.

## Results

### Genotype distribution

Among the 70 patients, 26 (37 %) had genotype *1/*1 (normal metabolizers). A heterozygous genotype carrying one loss of function allele (*1/*2 or *1/*3) was observed in 16 patients (~23 %), and two loss of function alleles (eg, *2/*2, *2/*3) were found in 17 patients (~24 %). Of these, specifically 6 patients (8.6 %) were homozygous *2/*2. The *17 allele, which can increase enzyme activity, was classified according to the predicted metabolizer phenotype.

### MiRNA expression by genotype

Analysis of five target miRNAs in three genotypic groups revealed some trends (Table 1).

Although none of the differences reached statistical significance, patients with genotype *2/*2 tended to have a higher relative expression of miR-126-3p and miR-185-5p compared to wild type *1/*1. Specifically, the *2/*2 group showed approximately 1.7 times higher expression of miR-126-3p (mean 1.72 ± 0.29 vs. 1.00 ± 0.18 in *1/*1; p=0.12) and 1.5 times higher expression of miR-185-5p (1.51 ± 0.24 vs. 1.00 ± 0.15; p=0.15). For miR-19a-3p, miR-19b-3p, and miR-22-3p, no significant differences between genotypic groups were observed (all p > 0.4). These data are summarized in Table 1.

In addition to group comparisons, linear regression analysis showed an insignificant inverse correlation between predicted CYP2C19 enzymatic activity and miR-126-3p levels. For every 10 % reduction in enzyme activity, miR-126-3p expression increased by an estimated 4.2 % (regression coefficient β = 0.42; p = 0.08). Similarly, miR-185-5p expression tended to increase with increasing number of *2 alleles (i.e., lower metabolizer status), although this did not reach significance. On the contrary, miR-19b-3p showed a slight and insignificant negative trend with more *2 alleles (β = −0.07; p = 0.45), and miR-22-3p showed stable expression in all genotypes (no genotype-dependent change).

### Clinical characteristics and phenotypic groups

We also investigated whether baseline clinical and laboratory parameters differed by the phenotype of the clopidogrel metabolizer. Patients were classified as normal or poor metabolizers according to their CYP2C19 genotype. The results of this analysis are presented in Table 2. There were no statistically significant differences in any of the clinical variables examined between the metabolizer groups. In particular, age, body mass index (BMI), lipid profile (total, high-density lipoprotein (HDL), low-density lipoprotein (LDL) cholesterol), blood count (hemoglobin, leukocyte count, platelet count, etc.), C-reactive protein (C-reactive protein (CRP)) and blood glucose levels were comparable between normal and poor metabolizers (p > 0.05 for all comparisons). This suggests that the groups of patients were clinically similar and, therefore, differences in miRNA expression are unlikely to be biased by these baseline factors.

Due to the small number of patients with *2/*2, the study power to detect a 1.5-fold difference in miRNA expression was limited. Post hoc power was calculated at only 38 % (for α = 0.05). Post hoc analysis of the sample size showed that approximately 42 patients in the *2/*2 group would be needed to achieve statistical significance of observed differences in expression, highlighting the need for a larger cohort to confirm these trends.

## Discussion

In future research, it would be beneficial to consider the immature platelet fraction (IPF) or other markers of platelet, formation, as these may correlate with the function of miR-126-3p and provide further information on platelet turnover.

Previous studies have explored miRNA expression in platelet function and clopidogrel response, including trends similar to our findings. Our data further support the hypothesis that miR-126-3p and miR-185-5p might serve as potential biomarkers of resistance to clopidogrel, although larger studies are necessary to validate this.

A limitation of our study is the lack of adherence evaluation, which could affect the interpretation of the clopidogrel response. This should be addressed in future studies. This study investigated the relationship between CYP2C19 polymorphisms and the expression of selected platelet microRNAs in patients with ischemic stroke treated with clopidogrel. We evaluated three genotypic groups (*1/*1, *1/*2, *2/*2) and the expression of five miRNAs (miR-126-3p, miR-19a-3p, miR-19b-3p, miR-22-3p, miR-185-5p) that are believed to have regulatory effects on platelet function.[Fig f1-pr74_669]

Although our results did not show statistically significant differences in miRNA levels between genotypic groups, we observed a trend towards higher expression of miR-126-3p and miR-185-5p in patients with genotype *2/*2 (poor metabolizers).

MiR-126-3p plays a key role in the regulation of vascular integrity and angiogenesis. This miRNA directly affects platelet function by modulating the expression of the ADAM9 and P2Y_12_ receptors, which are important molecules for platelet activation and aggregation [1,2]. The decreased expression of miR-126-3p has been associated with increased platelet reactivity, while increased expression may contribute to inhibition of platelet aggregation [1,2]. miR-185-5p mainly regulates the PI3K/Akt signaling pathway, which is crucial for platelet activation and response to various agonists [3]. Increased expression of miR-185-5p may suppress PI3K/Akt signaling and therefore reduce platelet reactivity [3].

Upregulation of these miRNAs in the group with genetically reduced clopidogrel activation (*2/*2) may reflect a compensatory mechanism or an alternative adaptive pathway to counteract reduced inhibition of P2Y_12_ [1,2]. In other words, platelet miRNAs such as miR-126-3p and miR-185-5p could increase in poor metabolizers as an epigenetic response to maintain platelet inhibition. However, this hypothesis requires further investigation, as our study was not sufficiently powered to confirm the differences statistically.

In contrast, the other miRNAs analyzed (miR-19a-3p, miR-19b-3p, miR-22-3p) did not show genotype-dependent changes in expression. This stable expression in all CYP2C19 genotypes suggests that these particular miRNAs are not affected by metabolic variants of CYP2C19. For example, miR-19b-3p showed a slight downward trend in carriers of *2 alleles, but this was not significant and is consistent with reports in the literature that do not associate miR-19b-3p with platelet function [5]. Similarly, miR-22-3p expression remained consistent regardless of genotype, suggesting that its regulation is likely independent of CYP2C19-related pathways [6].

Importantly, we found that clinical and laboratory characteristics were comparable between normal and poor metabolizers (Table 2). No significant differences in age, inflammatory markers, blood counts, or other comorbid factors were observed between the groups. This confirms that the observed miRNA expression patterns are primarily associated with genotype rather than external phenotypic or clinical variables. In other words, the trend toward higher miR-126-3p and miR-185-5p in poor metabolizers is likely a direct consequence of the genetic effect on clopidogrel metabolism rather than a confounding effect of baseline clinical status or comorbidities.

Our study has several limitations that need to be mentioned. The most significant limitation is the small number of patients with poor metabolism; only 6 individuals with genotype *2/*2 were included (post hoc power of ~38 % for α = 0.05), significantly limiting the statistical power [7]. Another limitation is the measurement of miRNA expression in plasma rather than isolated platelets. Plasma miRNA levels may not perfectly reflect the intracellular content of miRNAs in platelets and may be affected by contamination by other cells or exosomes [8,9].

## Recommendations for Future Research

We recommend that future studies include a larger number of patients, especially those with the *2/*2 genotype, in order to confirm the observed trends and achieve the necessary statistical power. Future investigations should also consider the analysis of miRNA expression in isolated platelets; this approach may provide more accurate information regarding intracellular miRNA levels and improve the interpretation of the results. Ideally, a multivariate analysis should be performed that incorporates possible confounders to further isolate the effect of the genotype on miRNA expression from other influencing variables. Furthermore we recommend expanding the discussion by incorporating the latest literature on the regulation of platelet functions by miRNAs and discussing the potential clinical implications of these findings, particularly in the context of personalized antiplatelet therapy. The implementation of these recommendations could significantly improve future studies and help validate the trends identified in this pilot research, thus contributing valuable insights to the fields of pharmacogenetics and neuropharmacology.

## Conclusions

This pilot study revealed a trend towards higher expression of miR-126-3p and miR-185-5p in patients with the CYP2C19 genotype *2/*2 treated with clopidogrel. These findings suggest a possible epigenetic regulation of the response to antiplatelet therapy, while the other miRNAs examined (miR-19a-3p, miR-19b-3p, miR-22-3p) appeared to be genotype-independent in their expression.

## Figures and Tables

**Fig. 1 f1-pr74_669:**
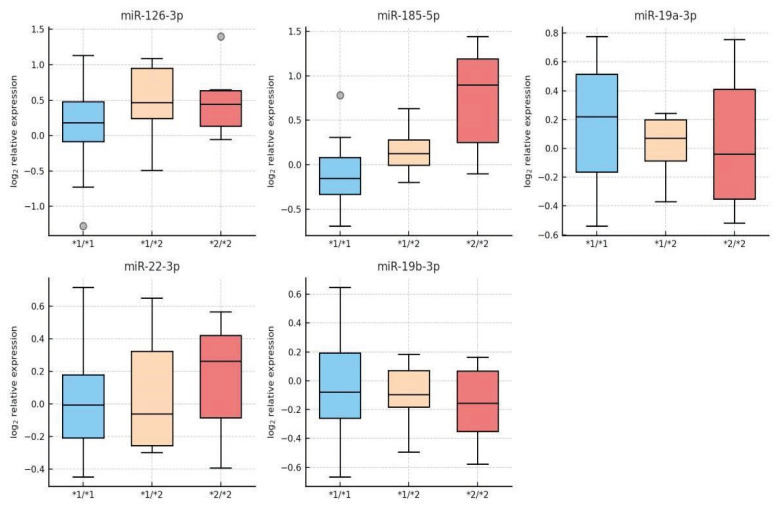
Box plots of log2-transformed miRNA expression levels demonstrate a trend towards higher expression of miR-126-3p and miR-185-5p in the *2/*2 genotype group, while miR-19a-3p, miR-19b-3p, and miR-22-3p show similar distributions between genotypic groups. The midline indicates the median, the boxes represent the interquartile range, and the whiskers indicate the range (excluding outliers).

**Table 1:** Quantitative analysis of platelet miRNA expression by the CYP2C19 genotype (relative expression ± SEM).

miRNA	Normal metabolizers (*1/*1), n=26	Intermediate metabolizers (*1/*2, *1/*3), n=16	Poor metabolizers (*2/*2, *2/*3), n=17	P-value
miR-126-3p	1.00 ± 0.18	1.32 ± 0.10	1.72 ± 0.29	0.12
miR-185-5p	1.00 ± 0.15	1.18 ± 0.09	1.51 ± 0.24	0.15
miR-19a-3p	1.00 ± 0.12	0.97 ± 0.14	0.89 ± 0.16	0.62
miR-19b-3p	1.00 ± 0.09	0.94 ± 0.10	0.87 ± 0.20	0.58
miR-22-3p	1.00 ± 0.10	1.05 ± 0.30	1.11 ± 0.50	0.41

Data are presented as mean ± SEM. Statistical analysis was performed using the Kruskal–Wallis test. Relative expression values are normalized to *1/*1 (set to 1.00). No comparisons reached statistical significance (p < 0.05; Kruskal–Wallis test).

**Table 2:** Phenotypic characteristics of patients by clopidogrel metabolizer status.

Parameter	Normal n=26	Intermediate n=16	Poor n=17	P – value
*Age (years)*	48.0 ± 10.0	49.0 ± 9.5	47.5 ± 8.8	0.6042
*Height (cm)*	172 ± 5	170 ± 6	169 ± 10	0.5108
*Weight (kg)*	75 ± 12	76 ± 14	80 ± 15	0.8012
*BMI (kg/m* *^2^* *)*	26.4 ± 3.5	27 ± 3.8	28 ± 4.2	0.8128
*Hemoglobin (g/dl)*	140 ± 12	138 ± 14	137 ± 11	0.7821
*Hematocrit (%)*	42 ± 3	41 ± 4	41 ± 3	0.8010
*Total cholesterol (mmol/l)*	4.8 ± 1.10	4.9 ± 1.20	4.7 ± 1.30	0.7532
*HDL cholesterol (mmol/l)*	1.2 ± 0.30	1.3 ± 0.40	1.2 ± 0.32	n.s.
*LDL cholesterol (mmol/l)*	2.0 ± 0.45	2.9 ± 1.00	2.8 ± 1.10	n.s.
*Leukocytes (×10* *^9^* */l)*	7.2 ± 1.0	6.9 ± 1.0	7.5 ± 1.0	0.7820
*C-reactive protein (mg/l)*	5 ± 2	6 ± 3	7 ± 4	n.s.
*Platelets (×10* *^9^* */l)*	248 ± 65	256 ± 72	250 ± 70	n.s.
*Erythrocytes (×10* *^12^* */l)*	4.7 ± 0.5	4.6 ± 0.6	4.5 ± 0.7	n.s.
*Glucose (mmol/l)*	5.6 ± 0.8	5.7 ± 0.9	6.1 ± 1.2	n.s.

Data are presented as mean ± SEM. Statistical analysis was performed using the Kruskal–Wallis test. No statistically (n.s.) significant differences were found between the metabolizer groups for any parameter (Kruskal–Wallis test for continuous variables).
